# Physical Activity Profile of School‐Aged Children Born Very Preterm and Its Association With Sleep

**DOI:** 10.1111/apa.70606

**Published:** 2026-05-26

**Authors:** Maham Hassan, Saskia Koller, Annkathrin Gies, Axel R. Franz, Johanna Catharina Brückner, Vadim Förster, Christian F. Poets, Christoph Randler, Cornelia Wiechers, Mirja Quante

**Affiliations:** ^1^ Department of Neonatology University Children's Hospital, Eberhard Karls University Tübingen Germany; ^2^ Department of Population‐Based Medicine Institute of Health Science, University Hospital Tübingen Tübingen Germany; ^3^ Center for Pediatric Clinical Studies University Children's Hospital, Eberhard Karls University Tübingen Germany; ^4^ Department of Biology University of Tübingen Tübingen Germany

**Keywords:** actigraphy, activity levels, preterm birth, sleep

## Abstract

**Aim:**

To describe the association between different physical activity and sleep parameters in school‐aged children born very preterm.

**Methods:**

A cross‐sectional analysis was done in a cohort of 6‐ to 10‐year‐old children born at < 32 weeks' gestation at the Department of Neonatology, University of Tübingen, Germany. Data were collected between May and November 2022 using actigraphy for sleep and activity, as well as patient records, questionnaires, and anthropometric measurements.

**Results:**

Two hundred and thirty five children (46% girls) were analysed at a median age of 8.0 (6.0–9.0) years. Daily sleep duration averaged 8.5 (SD: ±0.7) h. 24.7% achieved daily recommendations of 60 min moderate to vigorous physical activity (MVPA). Total physical activity, measured as counts/min, differed based on gestational age, with children born < 28 weeks' gestation exhibiting higher activity than those born at 28–32 weeks. Regression analysis showed one standard deviation (SD) increase in sleep efficiency associated with a 0.14 SD decrease in total physical activity (Standardized β: −0.14, CI: −0.26 to −0.02) and 37% lower odds of being in the higher MVPA group (OR: 0.63, CI: 0.47 to 0.83).

**Conclusion:**

Our study revealed important differences in some aspects of sleep and activity behaviour by gestational age that warrant future investigations.

## Summary

- Few studies have investigated associations between sleep and physical activity in children born preterm at school age.
- Sleep quality was inversely associated with total physical activity, with children born extremely preterm depicting higher total physical activity levels compared to those born very preterm.
- Future longitudinal studies should investigate physical activity and sleep traits in relation to behaviour in children born very preterm as they might be important targets for designing interventions in this population.

**Abbreviations**
AASM: American Academy of Sleep Medicine
ADP: air displacement plethysmography
BMI: body mass index
CPM: counts per minute
IQR: interquartile range
LPA: light physical activity
MVPA: moderate to vigorous physical activity
NREM: non‐rapid eye movement
PSG: polysomnography
SD: standard deviation
TST: total sleep time
WASO: wake after sleep onset
WHO: world health organization

## 1 Introduction

According to the latest available estimates, the rate of preterm birth is around 7.9% in the Western world, with 15% occurring before 32 weeks' gestation [1]. Many comparative studies show that school‐aged children who were born preterm have lower physical activity levels and sports participation than their term counterparts [2, 3, 4]. However, other studies only report minimal or no such differences in physical activity profiles among children born preterm [5, 6]. The main predicting variables showing significant associations to childhood physical activity, especially in children born preterm, are birth characteristics (sex, gestational age at birth, neonatal brain injury) [4, 7] and parental education [5, 7], as well as exercise capacity and respiratory function [7, 8]. Another important variable that needs to be considered in children born preterm is sleep. A recent systematic review reported lower sleep quality, more nocturnal awakenings, and larger proportions of Non‐Rapid‐Eye‐Movement (NREM) sleep in children born preterm compared to those born at term [9].

The interrelation of sleep and physical activity is worth studying in this population as disturbances in both behaviours can contribute towards long‐term adverse health consequences such as obesity and cardiometabolic risks, impaired cognitive and behavioural functioning, poor school performance, etc. [10, 11, 12, 13]. Prematurity itself can also lead to similar negative outcomes [14]. Infants born preterm have a higher risk of increased body fat percentage at term‐equivalent age [15], which may render them more susceptible to developing cardiometabolic diseases. Hence, these children tend to be at a uniquely higher risk of all the negative effects of poor sleep and physical inactivity than term‐born children. In general, disturbed sleep is also associated with more hyperactivity‐inattention, emotional difficulties, and peer problems [16, 17, 18], while higher levels of physical activity are actually associated with better psychological and school‐related well‐being [19].

There seems to be an inconsistent relationship between sleep and physical activity in children. While some data in children show higher physical activity associated with better sleep [20, 21], there is also evidence to the opposite [22]. A meta‐analysis of 47 studies in healthy children aged 3 to 13 years found little association between sleep and physical activity and reported high heterogeneity [23]. In adults, physical health prospectively predicted subjective sleep quality and vice versa [24]. There is also some evidence for a temporal link between morning physical activity and subsequent sleep duration [25] as well as between sleep and subjective well‐being [26, 27]. This association, however, has not yet received much interest in children born very preterm. A recent cross‐sectional study from Canada found limited associations between sleep‐disordered breathing symptoms and higher activity levels in 7–9‐year‐old children born extremely preterm [28].

One way to objectively measure both physical activity and sleep is actigraphy. In children, wrist‐worn actigraphy has been widely used and validated in research and clinical settings [29, 30]. It allows us to determine rest and activity patterns in the child's natural setting over several days [31, 32].

Considering how both physical activity and sleep are important in determining overall health of children born preterm and the inconsistency regarding their interrelationship in the available literature, our study aimed to objectively measure both physical activity and sleep parameters using actigraphy in a cohort of school‐aged children born very preterm in Germany. We also wanted to look into associations between physical activity profiles and sleep characteristics and determine differences by gestational age. Measuring daily physical activity and sleep characteristics of children born very preterm, specifically at early school age, adds to the existing evidence and examining their associations can help fill an important gap in research as well as provide insights into these important health‐related factors.

## 2 Methods

This study was a cross‐sectional analysis of baseline data gathered for a planned longitudinal cohort study of children born very preterm at the Department of Neonatology, University of Tübingen, Germany. Children were recruited at age 6–10 years and data were collected between May 2022 and November 2022.

### 2.1 Participants

Children born at < 32 weeks' gestation at University Women's Hospital, Tübingen, between 01/01/2012 and 31/10/2016 (*N* = 543) were identified and approached in 2022. Out of these, parents of 308 children could be successfully contacted and provided written informed consent and were enrolled in the study.

### 2.2 Inclusion Criteria

These were births before 32 weeks' GA, being born at Tübingen University Hospital between 2012 and 2016, age between 6 and 10 years at the time of recruitment, an ability to communicate in German and written informed consent given by both parents.

### 2.3 Exclusion Criteria

Children were excluded if they had died, could not be contacted, or their parents did not provide consent.

### 2.4 Variables

We determined information on gestational age at birth, along with birth weight, birth length and multiple births, as well as presence of bronchopulmonary dysplasia (defined as the need for supplemental oxygen or any respiratory support at 36 weeks postmenstrual age) and intraventricular haemorrhage (along with its grade) from hospital records. Presence of cerebral palsy was identified from 2‐year Bayley follow‐up records. *Z*‐score for birth weight and length were calculated based on WHO growth charts [33] using Perccalc (Paedsoft, Tuebingen, Germany). Data on parental demographics, that is, age and education, were collected using questionnaires. We determined the number of parents with high school level education depending on the type of school leaving certificate (“Abitur”) and also classified a university degree or PhD as higher education. The anthropometric measurements and calculation of body mass index (BMI) was done during the study visit. We also analysed body composition, including body mass, fat mass and fat free mass, using air displacement plethysmography (Bodpod). The digital scales of the Bodpod were used to measure body weight to the nearest 1 g. Children's height was measured to the nearest 0.1 cm using a stadiometer (Dr. Keller Längenmesstechnik‐GmbH, Limbach, Germany). Children's physical activity and sleep were objectively measured using actigraphy and subjectively through questionnaires. Parents were asked about how many days a week their child plays outside or participates in structured activity (i.e., any sport). The responses were categorized as rare (2 or fewer days per week) or frequent (3 to 7 days per week).

### 2.5 Actigraphy Protocol

We asked children to wear an actigraph (model wGT3X‐BT, ActiGraph, Pensacola, United States) with a triaxial accelerometer on their non‐dominant wrist, 24 h a day (except while bathing/showering or swimming/contact sports), for 1 week. It recorded children's activity in 60 s epochs. A sleep/wake diary of children's bedtime was also kept by parents during the same period. Data of returned actigraphs were downloaded using Actilife 6 Software, which translates the recorded accelerometer data into daily activity counts and uses these counts to determine periods of sleep and physical activity. An established protocol [11] was followed, that is, data for each day of recording were considered valid if more than 10 h of activity counts could be collected. If there were less than 5 days (or 4 nights) of valid data, families were asked to repeat the study. The Troiano algorithm 2008 was used for wear time validation [34]. The sleep/wake diary was used to determine the major sleep period of each study day as it has been recommended for use in actigraphy studies to help differentiate between sleep and the awake resting phase [35]. If there was a discrepancy of more than 30 min between Time‐to‐Bed/Out‐of‐Bed times between the sleep diary and the actigraphy, the latter data were used as final. As participants wore the actigraph continuously, total time in bed was subtracted from 24 h to get the awake time for physical activity determination.

### 2.6 Physical Activity Measurement

We scored physical activity from axis 1 using the Chandler algorithm in 60 s epochs [31]. We used three physical activity variables in this study: The number of counts per minute (CPM) from axis 1 averaged over the whole measurement period (i.e., 10 080 epochs or 7 days) gave the variable total physical activity, measured as CPM. It can be considered a summary measure that has been used previously in calibration studies [36]. The other two variables were light and moderate to vigorous physical activity per day (LPA, MVPA). The activity levels were determined using Chandler et al.'s cut points for axis 1 (i.e., 1932–6349 counts per minute = LPA and > 6349 counts per minute = MVPA) [31]. Time spent in LPA and MVPA everyday over the whole measurement period (7 days) was averaged to get the mean LPA and MVPA in minutes per day for each subject. The world health organization (WHO) recommends more than 60 min of MVPA per day for children and adolescents, which was used for grouping in analysis [37].

### 2.7 Sleep Parameters Measurement

Data from the same actigraph were also analysed for sleep and scored using the Cole‐Kripke algorithm [32]. We used the following sleep variables: (1) Sleep duration or Total Sleep Time (TST)—this is the total time in minutes spent in sleep as given by the algorithm—and converted to hours for analysis (hours/day). The American Academy of Sleep Medicine (AASM) recommends 9 to 12 h of sleep per night for children in this age range [38], so 9 h was used as a cut‐off to determine adequate sleep in our sample. (2) Time To Bed—this is the time of the day when the children went to bed. (3) Sleep Efficiency—this is the time actually spent asleep out of the total time spent in bed—given as a percentage by the algorithm. (4) Wake After Sleep Onset (WASO)—this is the time spent awake after sleep onset. It was not included in the total sleep time and was given in minutes by the algorithm. The latter two variables were considered to represent sleep quality. WASO is a parameter measured by actigraphy which can give a hint on sleep fragmentation. Data for these variables were averaged over the whole measurement period, that is, 7 nights.

### 2.8 Statistical Analysis

We tested all continuous variables for normality using Shapiro‐Wilkin test. Outliers were identified using boxplots for all variables, only the extreme outliers were discarded from final analysis. We grouped both, MVPA/day and TST/day, into binary variables based on recommended guidelines mentioned above. Continuous variables were described as means (with standard deviation) or medians (with interquartile range) based on their distribution, while data for categorical variables were given as number of subjects (with percentages). Continuous variables were compared using the independent sample *t*‐test or Welsh test. We used the chi‐square test to compare categorical variables. The difference of various sleep parameters across levels of activity (i.e., less or more than median of MVPA/day) was also determined using *t*‐test. Correlations between all activity and sleep variables were determined using Spearman's coefficient. Based on physical activity and sleep variables that showed significant correlations, we did regression analysis to further explore these associations. Three separate models were run, each using a different physical activity parameter as the dependent variable and the significantly correlated sleep variables as independent variables. Model 1 was simple linear regression of sleep efficiency (exposure) on total physical activity (outcome). In model 2, logistic regression was performed using MVPA categories as the outcome. MVPA was categorized into high and low physical activity using the median to get equal distributions in both categories. Model 3 used LPA/day as a continuous outcome while both sleep efficiency and Time To Bed were used as exposures. All models were adjusted for age, sex, gestational age at birth, body fat percentage and maternal education as these were considered to be important covariates [4, 39]. All continuous variables were Z‐standardized for regression analysis which gives us standardized beta coefficients in linear regression. All independent variables were checked for multicollinearity using variance inflation factor and residuals were checked for normality and homoscedasticity through visualizations. All analyses were done using the statistical software SPSS version 30.0.0.0.

## 3 Results

### 3.1 Participants Flow

**FIGURE 1:**
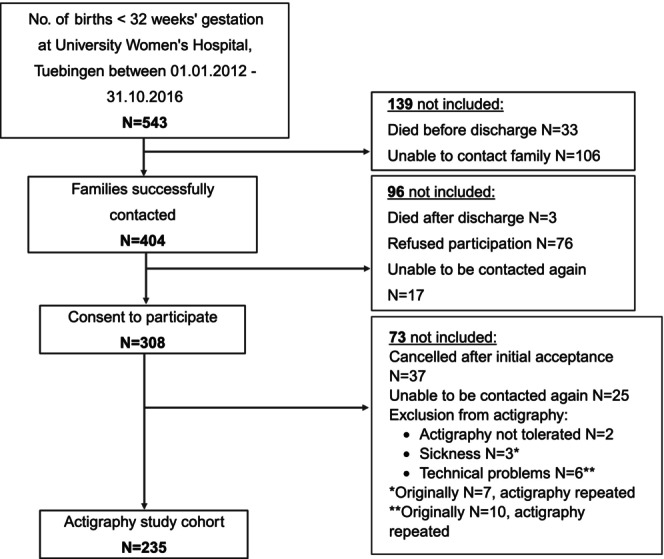
Flowchart of study participants.

Out of 543 eligible children born very preterm in Tübingen between 2012 and 2016, 308 (56.7%) could be successfully contacted and their parents gave consent for participation. Of these, 62 dropped out after initial consent (Figure 1). Two did not tolerate the actigraphy device, three became ill during its measurement and another six had technical problems. Actigraphy studies were repeated in eight children. The final analysis sample consisted of 235 children, that is, 43% of the original cohort. Baseline characteristics like gestational age and anthropometric data at birth and discharge were similar in the 510 preterm infants discharged alive compared to the 235 participants included in this analysis (Table 1).

**TABLE 1:** Infant characteristics of the total cohort compared to the children analysed in this study.

	All surviving infants with GA < 32 weeks (*N* = 510)	Study population (*N* = 235)	*p*
	Mean (±SD)/Number (%)		
Data at birth			
Gestational age, weeks	29.1 (±2.2)	28.8 (±2.2)	0.13
Sex, female	263 (51.6)	109 (46.3)	0.18
Number of multiple births	239 (46.8)	103 (43.8)	0.44
Birth weight, kg	1.1 (±0.4)	1.1 (±0.4)	0.49
Birth length, cm	37.0 (±4.4)	36.9 (±4.2)	0.71
Head circumference, cm	26.2 (±2.7)	26.0 (±2.6)	0.29
Children with bronchopulmonary dysplasia	39 (7.6)	14 (5.9)	0.40
Children with intraventricular haemorrhage (any grade)	32 (6.2)	13 (5.5)	0.97
Data at discharge			
Postmenstrual age, weeks	37.4 (±3.2)	37.3 (±3.4)	0.68
Discharge weight, kg	2.6 (±0.6)	2.6 (±0.6)	0.88
Discharge length, cm	45.2 (±3.9)	45.4 (±3.4)	0.44
Discharge head circumference, cm	32.4 (±2.3)	32.4 (±2.3)	0.89

### 3.2 Descriptive Characteristics

Children had almost equal sex proportions (46.3% female) and a median body fat percentage of 13.7% at a median age of 8.0 (IQR: 6.0; 9.0) years. While most children in this cohort were born after 28 weeks' gestation (65.5%), 81 (34.4%) were born extremely preterm, that is, at < 28 weeks' gestation (Table 2).

**TABLE 2:** Demographic characteristics of study participants.

	Total *N* = 235	Very preterm *N* = 154	Extremely preterm *N* = 81
	Median (Q1; Q3) or Mean (±SD) or *N* (%)		
Birth characteristics			
Birth weight, kg	1.1 (0.8; 1.4)	1.3 (1.1; 1.8)	0.8 (0.6; 0.9)
Birth weight *Z*‐score	−0.3 (±0.8)	−0.2 (±0.8)	−0.4 (±0.8)
Sex, female	109 (46.3)	69 (63.3)	40 (36.7)
Multiple births, *N* (%)	103 (43.8)	70 (45.5)	33 (40.7)
Bronchopulmonary dysplasia, *N* (%)	14 (5.9)	3 (2.0)	11 (14.2)
Intraventricular Haemorrhage, *N* (%)	13 (5.5)	3 (2.0)	10 (12.3)
Characteristics at study time			
Age, years	8.0 (6.0; 9.0)	8.0 (6.2; 9.0)	7.0 (6.0; 8.8)
Cerebral Palsy, *N* (%) (Missing = 26 subjects)	4 (1.7)	1 (0.7)	3 (4.2)
Weight, kg (Missing = 6 subjects)	24.2 (20.4; 28.8)	25.6 (21.2; 29.7)	23.1 (18.9; 26.7)
Weight WHO *Z*‐score (Missing = 6 subjects)	−0.3 (±1.2)	−0.2 (±1.2)	−0.5 (±1.1)
BMI standard deviation score (Missing = 6 subjects)	−0.64 (±1.3)	−0.6 (±1.3)	−0.7 (±1.2)
Body Fat Percentage, % (Missing = 6 subjects)	13.7 (9.9; 18.5)	13.5 (9.9; 19.1)	13.9 (8.9; 17.9)
Parental characteristics			
Mother's age, years	41.4 (±5.0)	41.9 (±4.9)	40.4 (±5.2)
Father's age, years (Missing = 3 subjects)	44.0 (40.0; 48.0)	45.0 (40.0; 48.0)	44.0 (39.0; 49.0)
Mothers with high school education (“Abitur”) (Missing = 3 subjects)	112 (47.7)	74 (48.6)	38 (47.5)
Fathers with high school education (“Abitur”) (Missing = 7 subjects)	111 (47.2)	74 (49.3)	37 (47.4)
Mothers with higher education (University Degree or PhD) (Missing = 8 subjects)	90 (39.6)	57 (38.5)	33 (41.7)
Fathers with higher education (University Degree of PhD) (Missing = 8 Subjects)	90 (39.6)	58 (38.6)	32 (41.5)

### 3.3 Physical Activity Variables and Their Distribution Across Degree of Prematurity

**FIGURE 2:**
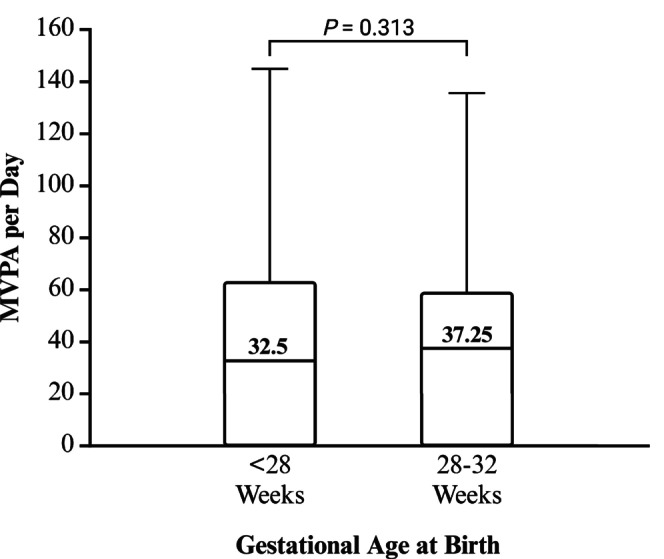
Difference in MVPA per day in extremely preterm and very preterm born children. MVPA, moderate to vigorous physical activity; *p* = *p*‐value.

**FIGURE 3:**
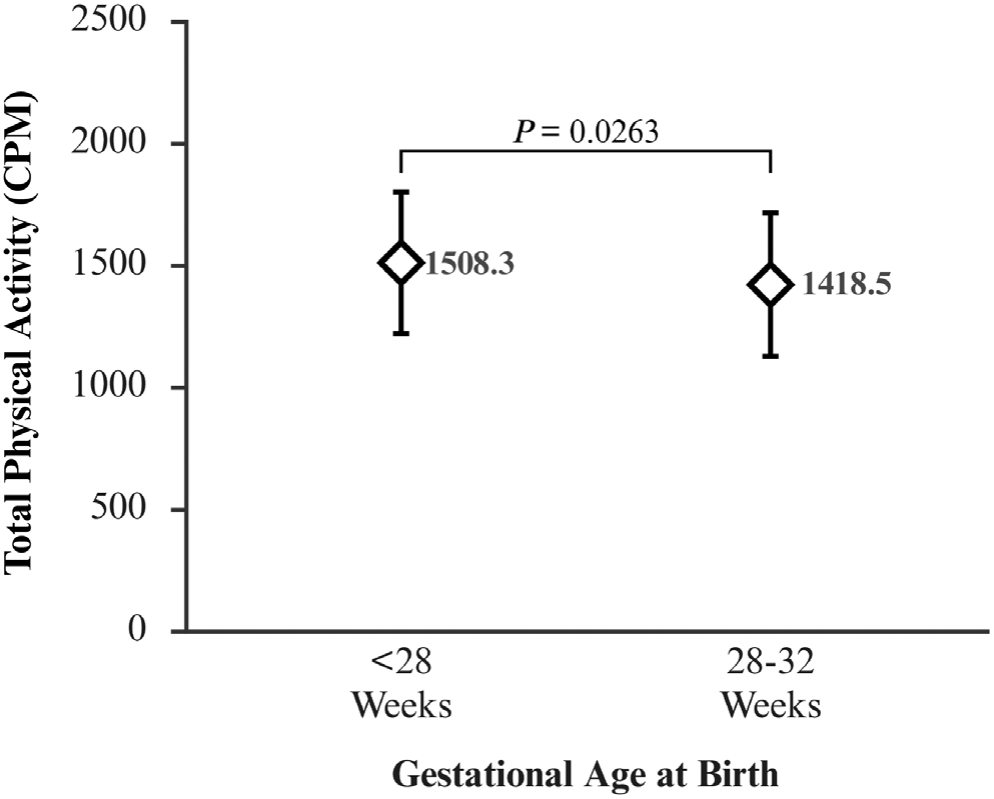
Difference in total physical activity between extremely preterm and very preterm born children. CPM, counts per minute; *p* = *p*‐value.

The median duration of MVPA/day in this cohort was 36.4 (IQR: 0.0; 59.9) minutes per day with only one quarter of children achieving the recommended daily levels of 60 min. MVPA per day differed by 5 min between children born before and after 28 weeks' gestation, although not statistically significant (Figure 2). However, the total physical activity was significantly higher in those born before 28 weeks' gestation (1508 ± 291 CPM) than those born after (1419 ± 294 CPM) (Figure 3). Further analysis indicated significant but weak negative correlations of total physical activity (Spearman *r*: −0.15) and LPA/day (Spearman *r*: −0.13) with gestational age (Table S1). In subgroup analysis, we did not see any major differences in physical activity patterns by bronchopulmonary dysplasia or intraventricular haemorrhage at birth (Table S2). Total physical activity also did not significantly differ by structured activity or time playing outside (Table S3).

### 3.4 Sleep Variables and Their Distribution Across the Degree of Prematurity

75.8% of children did not reach the recommended 9 h of sleep, although mean sleep duration (8.5 h) was only slightly below 9 h in this cohort. The mean sleep efficiency was also lower than the normal range of > 85% in these children (Table 3). Although both mean sleep efficiency and total sleep time were lower in children born before 28 weeks as compared to those born later, differences were not statistically significant.

**TABLE 3:** Physical activity and sleep parameters of participants.

	Total *N* = 235	Very preterm *N* = 154	Extremely preterm *N* = 81	*p*
Physical activity				
Total physical activity, counts/min	1449.5 (±295.1)	1418.5 (±293.7)	1508.3 (±290.6)	**0.02**
LPA/day, min	276.6 (±54.5)	269.9 (±531.3)	289.3 (±58.4)	**0.01**
MVPA/day, min	36.1 (±34.3)	37.7 (±33.9)	33.08 (±35.1)	0.33^a^
Children with MVPA > 60 min	58 (24.7)	37 (24.0)	21 (25.9)	0.74
Sleep				
Time To Bed, hour:min	21:08 (±00:50)	21:11 (±00:49)	21:04 (±00:52)	0.33
Sleep Efficiency, %	84.2 (±4.4)	84.3 (±4.3)	83.9 (±4.7)	0.48
Wake After Sleep Onset, min	82.7 (±27.2)	82.4 (±26.5)	83.4 (±28.6)	0.79
Total Sleep Time, hours/day	8.5 (±0.7)	8.5 (±0.7)	8.4 (±0.7)	0.07
Children with TST > 9 h	57 (24.2)	37 (24.0)	20 (24.6)	0.91

Abbreviations: BMI, body mass index; CPM, counts per minute; LPA, light physical activity; MVPA, moderate to vigorous physical activity; Q1, lower quartile; Q3, upper quartile; SD, standard deviation; TST, total sleep time.

^a^ *p*‐value from comparison of mean using Welsh test, rest are from independent *t*‐test, significant at 0.05 level.

### 3.5 Sleep Across Levels of Physical Activity

Children in the low (below median) MVPA group had significantly higher sleep efficiency and lower WASO when compared to those in the high (above median) MVPA group (Table 4).

**TABLE 4:** Difference in sleep parameters across levels of MVPA.

Sleep parameters	Moderate to vigorous physical activity categories			
	High (*n* = 118)	Low (*n* = 117)	*p*	Effect size
Time to bed (hours:min)	21:03 (±00:44)	21:13 (±0:55)	0.11	0.20
Sleep efficiency (%)	83.2 (±4.5)	85.2 (±4.2)	**< 0.001**	0.45
Wake after sleep onset (min)	87.3 (±28.3)	78.2 (±25.5)	**0.01**	0.34
Total sleep time (hours/day)	8.5 (±0.7)	8.5 (±0.7)	0.95	0.01

*Note:* Given as mean (SD), *p*‐value from Independent *t*‐test (two‐sided; significant at 0.05 level), Cohen's *D* effect size.

### 3.6 Correlation Between Sleep and Physical Activity Variables

We saw weak but significant negative correlations between sleep efficiency and all activity parameters. Time in LPA per day was also negatively correlated with Time To Bed and positively with WASO (*p*‐value < 0.05) (Table 5).

**TABLE 5:** Correlations between physical activity and sleep parameters.

	Physical activity parameters			Sleep parameters			
	Total physical activity	LPA	MVPA	Time to bed	Sleep efficiency	WASO	TST
Physical activity parameters							
Total physical activity	1.00	—	—	—	—	—	—
LPA	0.75^a^	1.00	—	—	—	—	—
MVPA	0.28^a^	0.09	1.00	—	—	—	—
Sleep parameters							
Time to bed	−0.10	−0.18^a^	−0.11	1.00	—	—	—
Sleep efficiency	−0.15^a^	−0.18^a^	−0.20^a^	0.15^a^	1.00	—	—
WASO	0.07	−0.14^a^	−0.11	−0.25^a^	0.91^a^	1.00	—
TST	−0.12	−0.10	0.00	−0.23^a^	0.38^a^	−0.13	1.00

*Note:* Spearman correlation coefficients.

Abbreviations: CPM, Counts per minute; LPA, light physical activity; MVPA, moderate to vigorous physical activity; TST, total sleep time; WASO, wake after sleep onset.

^a^ Significant at 0.05 level (two‐tailed).

### 3.7 Regression Analysis

Simple linear regression (model 1) using standardized variables indicated that one SD decrease in sleep efficiency was associated with a 0.14 SD increase in total physical activity (standardized beta coefficient: −0.14, CI: −0.26 to −0.02). Findings persisted after adjusting for age, sex, gestational age at birth, body fat percentage, and maternal education. Moreover, based on logistic regression (model 2), one SD decrease in sleep efficiency also increased the odds of being in the high MVPA group by 60% (Table 6).

**TABLE 6:** Regression of sleep parameters on physical activity parameters.

Sleep parameters	Physical activity parameters	
**Model 1**		
	Total physical activity	
	Standardized Beta (CI)	Significance
Sleep Efficiency	−0.14 (−0.26 to −0.02)	**0.02**
Adjusted *R* ^2^ = 0.171		
**Model 2**		
	High MVPA (above median group)	
	Odds Ratio (CI)	Significance
Sleep Efficiency	0.63 (0.47–0.83)	**0.001**
Nagelkerke *R* ^2^ = 0.132		
**Model 3**		
	LPA per Day	
	Standardized Beta (CI)	Significance
Sleep Efficiency	−0.16 (−0.28 to −0.04)	**0.006**
Time To Bed	−0.00 (−0.20 to 0.03)	0.18

*Note:* Adjusted *R* ^2^ = 0.160; *p*‐value significant at 0.05 level. Adjusted for age, sex, gestational age at birth, body fat percentage, maternal education.

Abbreviations: CI, confidence interval; CPM, counts per minute; LPA, light physical activity; MVPA, moderate to vigorous physical activity.

Regression analysis of significantly correlated sleep variables with LPA (model 3) showed that sleep efficiency was also negatively associated with time spent in LPA, while Time To Bed was not a significant predictor of LPA/day after adjustment (Table 6). The complete regression models with all included variables are presented in the Supporting Informations (Table S4).

## 4 Discussion

This study aimed to analyse physical activity and sleep characteristics of 6‐ to 10‐year‐old children born very preterm. Our results show that on average, age‐specific international recommendations for daily MVPA and sleep duration were not met in this cohort. There were significant gestational age‐based differences in physical activity profiles among these children. Moreover, sleep efficiency was negatively associated with physical activity levels (both LPA/day and MVPA/day), and children with lower sleep efficiency also had higher total physical activity.

Our findings are in contrast to the EXPRESS cohort in Sweden, where average actigraphy‐measured MVPA (mean = 90 min/day) in 6.5 year‐old extremely preterm children (*N* = 71) was above the recommended level of 60 min/day [4]. Similarly, male very preterm (< 32 weeks) children (*N* = 79) from the Millenium Cohort in the UK also met these recommendations at 7 years of age when assessed objectively using actigraphy (mean MVPA = 61 min/day) [7]. However, two other actigraphy‐based studies from the UK showed that preterm children at an age of around 11 years did not achieve recommended MVPA levels, similar to our results [6, 40]. These differences suggest that achieving recommended MVPA levels might be an issue at later ages in children born preterm. However, as our cohort, although large, is only monocentric and hence not representative of the underlying population, generalizations about German children born preterm cannot be made.

Interestingly, our results showed significantly higher total physical activity in children born extremely preterm (< 28 weeks) as compared to those born at 28–32 weeks. On the other hand, no such differences were seen between MVPA levels of children born extremely vs. very preterm. The higher total physical activity, measured as counts, in extreme prematurity could point towards increased motor activity and depict the association of extreme prematurity with a high activity trait, which has been reported before [41].

Sleep duration in our cohort was almost reaching recommended levels with a mean duration of 8.5 h. This was similar to estimates of other studies using subjective and objective assessments [9]. The slightly lower than normal mean sleep efficiency (84.2%) in our cohort was in contrast to two other studies (*N* = 58 and *N* = 85) that showed a mean sleep efficiency of > 90% in 6 to 12 year old children born preterm using polysomnography (PSG) [18, 42], but in line with actigraphy‐studied cohorts from Canada (*N* = 101) and Australia (*N* = 87) that also showed sleep efficiencies of < 85% (mean = 79% and 81%, respectively) in 5‐ to 10‐year‐old children born very preterm [43]. In addition, Gössel‐Symank et al. revealed lower sleep efficiency in children born preterm (*N* = 17) compared to term‐born children (*N* = 8) at 20 months of age [13]. These differences in sleep efficiency of children born preterm might be due to different assessment methods, but even studies using the gold standard of PSG reported overall poorer sleep quality in children born preterm.

Another important finding of our study was that lower sleep efficiency was associated with increased total physical activity and greater odds of being in the higher MVPA group, which contradicts the usually positive effects of physical activity on sleep quality [20, 21, 44].

One possible explanation for the negative association of sleep quality with total physical activity could be the activity trait of personality [45], that is, children who show higher activity during the day are also more active at night and hence show poorer sleep. Actually, poor sleep–wake regulation has been established as one of the adverse neurological outcomes in infants born preterm [46] and these results could indicate that, even at later ages, children who are more active during the day might have greater issues regulating arousals at night time and hence show poorer sleep efficiency.

Another possible reason could be the presence of reverse associations as our data are cross‐sectional. Hence, it might be that children having poor sleep are actually showing motor restlessness, or even hyperactivity during the day. This is especially important to consider in a preterm cohort, since these children are known to have a higher incidence of Attention‐Deficit Hyperactivity Disorder (ADHD) [47]. Generally, children with ADHD tend to show more sleep disturbances [48] and one study actually established the mediating role of prematurity between hyperactivity/impulsivity symptoms and sleep disturbance of children with ADHD [16]. Moreover, a meta‐analysis of 24 studies (2179 children) confirmed higher total physical activity (also measured as counts by actigraphy) in children with ADHD compared to normally developing children and also showed evidence of altered sleep [49]. However, since ADHD symptoms were not recorded in our study, such causalities cannot be determined. Actigraphy can measure increased physical activity correlating to motoric hyperactivity but cannot measure inattention behavior. Of note, also sleep efficiency is not an entirely straightforward parameter, as it is greatly influenced by bedtime. If parents do not send their children to bed according to their sleep needs, for example, this can have a negative impact on sleep efficiency. Although we only had weak associations and moderate effect sizes, it could lead the way to future research on the dynamic association of sleep and physical activity in children born preterm using larger sample sizes and more robust analyses.

Using actigraphy to measure children's activity and sleep improves ecological validity of our assessment as children are in their natural environment and data are objective. Although actigraphy is a validated tool in sleep and activity research, it has certain limitations. Poor specificity has been reported for determining WASO, as well as a lack of standard scoring rules or variable definitions [50]. Moreover, in our study, we used 60 s epochs which can impact the amount of physical activity recorded especially in younger cohorts [51]. The large cohort of preterm born children is another strength, but as the cohort is only based in one center, generalizations about German children cannot be made. Our study encourages larger, multicenter studies of this kind to determine actigraphy measured sleep and physical activity profiles of children born preterm including systematic behavioural assessments.

Our study has several limitations. Since it is cross‐sectional, no inference on causation can be derived. Another limitation is the non‐availability of data on behavioural ADHD assessments and physical strengths of these children as well as parents' behaviours towards their activity, as these could also impact the physical activity profiles of these children. Moreover, it should be noted that this cohort comprises of a high proportion of parents with a high level of education, potentially indicating higher socioeconomic status as well. It might not be representative of other cohorts born preterm, as both physical activity and sleep in children are affected by parental education [5, 52], the results should be interpreted in light of this unique feature of our cohort. In twins or triplets, outcome variables may be correlated. In studies on preterm infants addressing neonatal outcomes and 2‐year outcomes it has been repeatedly shown that the intra‐family correlation is very poor (see for example [53]) – and, consequently, the twin intra‐class correlation coefficient was not considered for the analyses in this trial. Admittedly, we do not know if sleep and physical activity at 6 to 10 years may correlate in multiples. We choose to analyse our activity data using a 1‐min epoch setting since most published studies regarding physical activity and sleep are based on data collected using a 1‐min epoch setting. However, we agree that the choice of epoch length can affect the information content of activity data. Moreover, PSG, not actigraphy, is the gold standard to measure sleep. Since actigraphy relies on the absence of movement to estimate sleep without any corroborating physiological data (e.g., electroencephalography) or direct observation of behaviour, it is inevitable that there will be differences between actigraphy and PSG [50, 54]. For example, actigraphy identifies sleep onset before PSG and thus overestimates total sleep time and sleep efficiency [55].

In conclusion, the association of objectively measured physical activity profiles and sleep of former preterm children suggests that sleep health might play a specific role in modulating their activity behaviour or vice versa. Overall, our study implies a need for future longitudinal studies with systematic assessment of both physical activity and sleep in relation to behavioural testing.

## Author Contributions

**Saskia Koller:** investigation, data curation. **Annkathrin Gies:** investigation, data curation. **Johanna Catharina Brückner:** investigation, data curation. **Vadim Förster:** investigation, data curation. **Axel R. Franz:** conceptualization, methodology, writing – review and editing. **Maham Hassan:** writing – original draft, formal analysis, visualization. **Cornelia Wiechers:** conceptualization, methodology, project administration, writing – review and editing, funding acquisition. **Christoph Randler:** conceptualization, methodology, writing – review and editing. **Christian F. Poets:** conceptualization, methodology, writing – review and editing. **Mirja Quante:** conceptualization, methodology, supervision, funding acquisition, writing – original draft, project administration.

## Funding

This work was supported by Medizinischen Fakultät, Eberhard Karls Universität Tübingen (Grant 491‐0‐0 and 531‐0‐0).

## Ethics Statement

The institutional review board approved the study protocol (approval number 103/2022BO2) and written informed consent was obtained from parents. The study was registered in the German Clinical Trials Register (trial number DRKS00029386). It was conducted in accordance with the tenets of the Declaration of Helsinki regarding confidentiality of the data collected. Parents or legal guardians of children who agreed to participate signed a written informed consent form.

## Conflicts of Interest

This study was sponsored by the Medical Faculty of the University of Tuebingen through the Applied Clinical Research Program (AKF), grant number 491‐0‐0. Dr. Wiechers and Dr. Quante were supported by scholarships of the Medical Faculty of the University of Tuebingen (Tuebingen Program for the Advancement of Women in Science (TüFF) [CW] and Tuebingen Advanced Clinician Scientist Program, grant no. 531‐0‐0 [MQ]). The other authors report no conflicts of interest.

## Supporting information


**Table S1:** Correlation of gestational age at birth with physical activity.
**Table S2:** Physical activity and sleep parameters in children with and without the birth history of bronchopulmonary dysplasia and intraventricular haemorrhage.
**Table S3:** Total physical activity across frequency of sports or outside play.
**Table S4:** Complete adjusted regression models.

## Data Availability

The data that support the findings of this study are available from the corresponding author upon reasonable request.
