# Supplementary material for: Physical Activity Profile of School‐Aged Children Born Very Preterm and Its Association With Sleep
Source: Acta Paediatr. 2026 May 26;115(9):2018–28. doi: 10.1111/apa.70606 (PMC13460530; doi:10.1111/apa.70606)
Supplement: Supplementary file 1 [file APA-115-2018-s001.docx]

**Table S1:** Correlation of Gestational Age at Birth with Physical Activity

|  | Total physical activity | LPA/day | MVPA/day |
| --- | --- | --- | --- |
| Gestational Age at Birth | -0.15* | -0.13* | 0.01 |

Abbreviations: LPA=light physical activity; MVPA=moderate to vigorous physical activity

Spearman Correlation Coefficients; *Significant at 0.05 (two-tailed)

**Table S2:** Physical activity and sleep parameters in children with and without the birth history of bronchopulmonary dysplasia and intraventricular hemorrhage

|  | **Bronchopulmonary Dysplasia** | | | **Intraventricular**  **Hemorrhage** | | |
| --- | --- | --- | --- | --- | --- | --- |
|  | Yes  (N=14) | No  (N=212) | p-value | Yes  (N=13) | No  (N=222) | p-value |
| **Physical activity parameters** | | | | | | |
| Total Physical activity, counts/min | 1384.5  (±278.5) | 1453.9  (±299.1) | 0.40 | 1491.1  (±391.8) | 1447.0  (±289.4) | 0.60 |
| LPA/day, mins | 276.5  (49.0) | 275.8  (±55.1) | 0.96 | 290.9  (±63.1) | 275.7  (±53.9) | 0.41 |
| MVPA/day, mins | 36.1  (±32.2) | 36.1  (±34.5) | 0.99* | 45.9  (±39.7) | 35.5  (±33.9) | 0.28* |
| **Sleep parameters** | | | | | | |
| Time To Bed, hour:min | 21:03  (±00:49) | 21:09  (±01:11) | 0.14 | 20:33  (±00:56) | 21:10  (±00:49) | **0.01** |
| Sleep Efficiency, % | 85.8  (±3.7) | 84.0  (±4.5) | 0.18 | 83.9  (±3.5) | 84.2  (±4.5) | 0.80 |
| Wake After Sleep Onset, min | 73.4  (±24.2) | 83.4  (±27.7) | 0.70 | 87.8  (±21.3) | 82.4  (±27.5) | 0.48 |
| Total Sleep Time, hours/day | 8.6  (±1.0) | 8.4  (±0.7) | 0.36 | 8.9  (±0.6) | 8.4  (±0.7) | **0.008** |

Abbreviations: LPA=light physical activity; MVPA=moderate to vigorous physical activity, *p-value from comparison of mean using Welsh test, rest are from independent t-test

**Table S3:** Total physical activity across frequency of sports or outside play

|  | Playing outside | | | Sport frequency | | |
| --- | --- | --- | --- | --- | --- | --- |
|  | Rare  (N=7) | Frequent  (N=219) | p-value | Rare  (N=162) | Frequent  (N=61) | p-value |
| Total Physical Activity | 1343.5 | 1443.8 | 0.37 | 1428.8 | 1487.5 | 0.18 |
| MVPA/day | 18.5 | 36.3 | 0.21* | 32.5 | 44.9 | **0.02*** |
| LPA/day | 235.3 | 276.6 | 0.29 | 276.5 | 272.9 | 0.69 |

Abbreviations: LPA=light physical activity; MVPA=moderate to vigorous physical activity, *p-value from comparison of mean using Welsh test, rest are from independent t-test

**Table S4:** Complete adjusted regression models

| SLEEP PARAMETERS | PHYSICAL ACTIVITY PARAMETERS | |
| --- | --- | --- |
| **Model 1** | | |
|  | Total Physical Activity | |
|  | Standardized Beta (CI) | Significance |
| Sleep Efficiency | -0.14 (-0.26 to -0.02) | **0.02** |
| Age | -0.11 (-0.24 to 0.01) | 0.08 |
| Gender (male) | 0.39 (-0.02 to 0.80) | 0.06 |
| Gestational age at birth | -0.09 (-0.21 to 0.02) | 0.13 |
| Body fat percentage | -0.27 (-0.41 to – 0.13) | **<0.001** |
| Maternal education (low) | -0.09 (-0.28 to 0.48) | 0.61 |
| Adjusted R square = 0.171 | | |
| **Model 2** | | |
|  | High MVPA  (above median group) | |
|  | Odds Ratio (CI) | Significance |
| Sleep Efficiency | 0.63 (0.47 to 0.83) | **0.001** |
| Age | 1.54 (1.14 to 2.08) | **0.005** |
| Gender (male) | 0.68 (0.39 to 1.21) | 0.19 |
| Gestational age at birth | 1.00 (0.76 to 1.32) | 0.96 |
| Body fat percentage | 0.82 (0.60 to 1.12) | 0.22 |
| Maternal education (low) | 1.33 (0.75 to 2.36) | 0.32 |
| Nagelkerke R square = 0.13 | | |
| **Model 3** | | |
|  | LPA per Day | |
|  | Standardized Beta (CI) | Significance |
| Sleep Efficiency | -0.16 (-0.28 to -0.04) | **0.006** |
| Time To Bed | -0.00 (-0.20 to 0.03) | 0.18 |
| Age | -0.32 (-0.45 to -0.19) | **<0.001** |
| Gender (male) | 0.18 (-0.21 to 0.59) | 0.36 |
| Gestational age at birth | -0.08 (-0.21 to -0.03) | 0.15 |
| Body fat percentage | -0.09 (-0.22 to -0.04) | 0.19 |
| Maternal education (low) | 0.15 (-0.21 to 0.53) | 0.41 |
| Adjusted R squared = 0.174 | | |

Abbreviations: LPA=light physical activity; MVPA=moderate to vigorous physical activity
